# Reducing the Weight of Spinal Pain in Children and Adolescents

**DOI:** 10.3390/children8121139

**Published:** 2021-12-05

**Authors:** Thorvaldur S. Palsson, Alessandro Andreucci, Christian Lund Straszek, Michael Skovdal Rathleff, Morten Hoegh

**Affiliations:** 1 Department of Health Science and Technology, Faculty of Medicine, Aalborg University, 9220 Aalborg, Denmark; clns@hst.aau.dk (C.L.S.); misr@hst.aau.dk (M.S.R.); msh@hst.aau.dk (M.H.); 2 Center for General Practice at Aalborg University, 9220 Aalborg, Denmark; aa@dcm.aau.dk; 3 Department of Physiotherapy, University College of Northern Denmark, 9800 Aalborg, Denmark

**Keywords:** spinal pain, adolescent back pain, self-management

## Abstract

Spinal pain in adults is a significant burden, from an individual and societal perspective. According to epidemiologic data, spinal pain is commonly found in children and adolescents, where evidence emerging over the past decade has demonstrated that spinal pain in adults can, in many cases, be traced back to childhood or adolescence. Nevertheless, very little focus has been on how to best manage spinal pain in younger age groups. The purpose of this article is to put the focus on spinal pain in children and adolescents and highlight how and where these problems emerge and how they are commonly dealt with. We will draw on findings from the relevant literature from adults to highlight potential common pathways that can be used in the management of spinal pain in children and adolescents. The overall focus is on how healthcare professionals can best support children and adolescents and their caregivers in making sense of spinal pain (when present) and support them in the self-management of the condition.

## 1. Introduction

The burden of spinal pain has pandemic proportions, and it is the leading cause of disability in adults [1]. Now, evidence clearly points to the fact that long-lasting spinal pain seems to start in childhood or adolescence and is associated with an increased risk of spinal pain later in life [2,3,4]. There is no single cause for non-specific spinal pain but, in most cases, it is related to several biomedical, psychological, and social factors [5].

Depending on the definition of spinal pain, the condition affects somewhere between 6–39% of children and adolescents annually [6,7,8], where the prevalence rates and care-seeking behavior seems to gradually increase from childhood until late adolescence/early adulthood [9,10]. In addition to seeking care from healthcare professionals, the age group commonly resorts to passive approaches, e.g., using pain killers for self-management, as up to 30% of adolescents use pain medication on a weekly basis [11]. Seemingly, this strategy does not only stem from the need to reduce pain but also a means of managing emotional distress [12]. The multifactorial nature of pain is, in fact, evident when considering that those who use pain medication on a regular basis, have signs of widespread pain, report poor sleep quality, have lower self-esteem and are frequently absent from school [13,14]. Although this does not oppose the use of pain medication when indicated, it does suggest the need for a management strategy that encompasses the multiple domains potentially involved, where pain is just a part of the problem. Such strategies, e.g., dependency on healthcare professionals or pain medication for pain relief, are known to predict greater risk of sick leave in adults with back pain [15]. Based on the above, it may seem that non-specific spinal pain in children and adolescents is managed either passively or not at all.

Children and adolescents are not small adults. However, very little evidence exists of how to best manage spinal pain among the youngest patients. For this reason, we summarize the relevant research findings and examine whether knowledge thereof can inform management approaches in children and adolescents. We will draw on themes that are commonly focused on in the management of spinal pain in adults. The aim is to highlight some of the knowledge available from the literature from non-specific spinal pain and, based on this, suggest future directions to promote self-management of spinal pain amongst children and adolescents.

## 2. Understanding the World through the Eyes of Adults—The Role of Unhelpful Messaging

It is human nature to seek an underlying cause of a pain problem [16]. In the parent–child relationship, an initial assessment and management strategy is often (and rightfully so) conducted by the parent or guardian. In this regard, however, it is important to acknowledge that several misconceptions exist in relation to spinal pain in adult populations. One is the assumed relationship between biomechanical loading (e.g., posture) and spinal pain. Adults often perceive posture as being an important factor in their back pain [17], where extended, upright positions are generally considered the most correct way of sitting [17,18]. Interestingly, however, it seems that spinal pain is more strongly associated with, for example, emotional distress and conduct problems [19] than the spinal curvature in children and adolescents [20,21,22]. Experimental studies have shown that when compared with asymptomatic controls, adults with back pain tend to have decreased [23,24] or excessive [25] movement variability, generate excessive force via their trunk muscles when performing low-load tasks [26,27] and continue to use the same strategy when fatigue sets in in the trunk muscles [28]. It may therefore seem counterintuitive to promote certain postures when it seems to be the strategy to maintain the posture, which is the problem instead of the posture itself. Therefore, healthcare professionals should encourage children and adolescents to adopt comfortable postures (irrespective of spinal curvature) with reassurance that sitting in various ways is safe and may in fact provide symptom relief [29].

Often, prolonged sitting in front of a computer or using a smartphone, are considered the culprits when trying to find explanations for spinal pain in children and adolescents [30,31]. Although it may be tempting to jump to such conclusions, it is important to consider what the child is not doing instead of playing videogames or using the smartphone, i.e., being physically active. Another example is school bags (their weight, shape, and design). Often the recommendations regarding the choice of school bags are based on the weight of the bag not exceeding 10% of the child’s body weight, although a recent meta-analysis [32], including data from over 9000 children, did not find evidence to support this claim. Furthermore, Haselgrove et al. [33] found that adolescents who carried their schoolbag for more than 30 min per day, but used an inactive transportation method (e.g., were driven or took the bus) were more likely to report spinal pain than those using an active way of getting to school (walking/cycling), even though they carried their schoolbag for an equally long time. The underlying theme, therefore, seems to be that inactivity, rather than posture and load, appears to be an important contributing factor to spinal pain in children and adolescents.

There is convincing evidence suggesting that physical activity may reduce the risk of recurrent episodes or chronic spinal pain in adults [34] and may, therefore, serve as “protection” against the pain condition. This may relate to both physiological tissue adaptations to the exercise and also less fear/worrying of getting hurt, as transient soreness/fatigue is a common part of physical activity and exercise. A relationship between lower physical activity levels in adolescents and higher risk of pain specific to the spine [35], and in general [36], seems to exist, indicating that this may be a significant contributing factor to spinal pain in this age group. With this in mind, introducing more physical activity into everyday life, e.g., based on WHO’s recommendations (www.who.int/news-room/fact-sheets/detail/physical-activity (accessed on 12 July 2021)), instead of banning certain activities, may be a more viable and sustainable strategy to manage the negative aspects (including pain) of a sedentary lifestyle.

In summary, the vast majority of all spinal pain is benign and multifactorial, and, for adolescents, it is not abnormal to experience discomfort from sustained postures and/or carrying heavy loads. Importantly, the discomfort does not equate to an injury [37], nor does it pose any risk of future damage, although data suggests that pain is a recurrent condition for most people. From adult populations, we know that physical activity has the capacity to reduce spinal pain, or the burden of spinal pain, and it seems possible that this is also true for adolescents.

## 3. Attributing Musculoskeletal Symptoms to Damage—What Can We Learn from Adult Populations?

Back pain is commonly considered a result of damage to the back due to the flawed belief that the back is fragile and needs to be protected [38,39,40,41]. Studies investigating patients’ views of their own condition, have shown that adults who harbor pathoanatomical views of their spinal pain, report higher levels of disability [42]. In fact, it seems that difficulty in abandoning biomedical beliefs related to back pain is a barrier for recovery [43]. It is understandable that patients seek answers from healthcare professionals regarding underlying reasons for their pain, especially when it persists [44]. However, in spinal pain, as in most other pain conditions, a certain level of diagnostic uncertainty exists where the underlying cause cannot be identified, despite common beliefs hereof [45]. If the explanations provided are biased towards a potential underlying pathology and/or weakness, they may negatively affect the prognosis by promoting movement-related fear [46,47,48]. Indeed, psychological, and social factors are known to play important roles in the development and persistence of pain [49,50,51]. Moreover, children’s thoughts and beliefs, about pain are influenced by the interpretations of parents and other family members [52,53]. Parents/caregivers, as well as the child/adolescent, can struggle with the social consequences of persistent or intense episodic spinal pain, such as despair, fear and distress related to both stigma (e.g., “legitimacy” of a pain-related diagnosis) [54] and the uncertainty of how the disease will influence the future life of child and adolescent patients [55]. Often, the dominant belief is that spinal pain has a particular medical explanation, with less focus on emotional and behavioral factors [56]. With this in mind, it is evident that all strategies aimed at supporting recovery need to address child and parent/caregiver thoughts and beliefs regarding the underlying cause, prognosis and means of management. In adults, the risk of poorer outcomes increases if the psychological and social aspects of spinal pain are left unmanaged [57,58,59]. Furthermore, they seem to mediate disability after an initial episode of spinal pain in adults [60,61,62]. It is therefore equally important to address these factors in children and adolescents affected by spinal pain.

Based on the above, several factors (alone and in combination) can contribute to persistent spinal pain in children and adolescents. As for the parent/caregiver, this highlights the need to address the spinal pain as a lived experience, i.e., to understand how pain affects the child/adolescent and what values they attribute to the pain. Furthermore, it seems that education regarding the nature of spinal pain is important and may in fact be most beneficial for children and adolescents experiencing their first episodes of spinal pain [63]. Patient education on spinal pain should address misconceptions about the relationship between pain and tissue damage, and provide coherent, contemporary explanations of the multifactorial nature of spinal pain. Moreover, as spinal pain is currently considered an episodic condition [5], advice on self-management strategies to reduce or control future pain episodes should be included as first line treatment options [5]. In line with this, a four-step model was recently suggested [64] with the aim of guiding clinicians in the management of non-specific musculoskeletal pain conditions. Table 1 illustrates how this model is adapted to spinal pain in children/adolescents.

## 4. Moving Forwards—Strategies to Modify the Trajectory

In the current management of back pain, several evidence-based national guidelines exist to support clinical decision making [65] for the treatment/management of spinal pain. Rightfully so, as it gives clinicians freedom to choose and adapt interventions to the individual. In that respect, evidence favors advice, education, exercise (supervised and unsupervised) and cognitive behavioral therapy for spinal pain, whereas pharmacotherapy, manual therapy and other (commonly used) therapies are considered second-line or adjuncts [5]. However, no interventions seem to have a superior effect [66]. These guidelines are based on findings from adult populations, which is why any given approach needs to be appropriately adapted to the child/adolescent. It is important to note that the prognosis is generally good, even though people are left to self-manage [67]. Nevertheless, the onset of spinal pain is not always followed by a gradual return to normal as various patterns of recovery/persistence of pain are seen across individuals [68,69]. Therefore, deciding which intervention to use can only be carried out following a thorough assessment, including identification of the potential underlying driver(s) of the pain condition. Here, clinicians need to be mindful of not getting in the way of recovery and that the management strategy focuses on promoting self-management, instead of passive dependency on, for example, healthcare providers and/or medication.

## 5. Aligning Clinical Messaging to Contemporary Pain Science

Several processes contribute to the pain experience [70]. For example, acute damage to the structures of the spine inevitably involves the nociceptive system, conveying signals from the periphery to the central nervous system. But there is more to it than just nociceptive signaling [71]. As with other pain conditions, people of all ages who experience spinal pain try to make sense of their pain by drawing on previous experiences, and social and cultural contexts. A poorer outcome can be expected amongst those who believe their back pain will persist, have a perception of serious consequences and experience limited control of their pain [72]. In contrast, adults who succeed in reducing pain-related fear and gaining more control over their back pain, attribute this to an ability to be able to make (more) sense of their pain [73]. Explicitly, this indicates that by gaining insight into your own condition, what it (the pain) means, what causes the pain and future perspectives, this helps people to self-manage their pain condition. Directly extrapolating these factors from adult populations to children and adolescents may, however, not be possible as the cognitive skills, previous experiences, learning and social contexts (e.g., work) are considerably different between adults and younger populations. However, spinal pain remains influenced by the child’s social context and culture and may, to some degree, substitute their own lack of experience (e.g., being dependent of their surroundings to provide the information needed).

The common sense model [74] describes five broad dimensions that adults use to give meaning to a pain experience [73]:

(i) The identity of the pain—“*Where is my pain coming from?*”
(ii) The cause of the pain—“*Why does my back hurt?*”
(iii) What are the consequences of my pain?—“*How will the back pain affect my life?*”
(iv) How well can I control my pain?—“*What can I do to make it go away?*”
(v) How long will the pain last?—“*When will it stop?*”

Although not designed to replace subjective assessment in a clinical context, the model could form the backbone of the interview with the child/adolescent and thus provide insight into the lived experience of pain. This insight may then serve as a reference point for the patient’s education, which needs to address individual thoughts, beliefs, and emotions. An example of how the adolescent can be supported in making sense of their back pain is provided in Table 2.

## 6. Supporting Children and Adolescents in the Self-Management of Spinal Pain

Considering the prevailing belief amongst the general public that the back is fragile and that pain is related to a structural flaw [38,39,40,41], it is clear that any intervention addressing spinal pain in children/adolescents, needs to involve the parents/caregivers. A parent’s lack of understanding and/or appreciation of emotional and cognitive factors in relation to pain, may limit the acceptance of a multimodal approach, potentially resulting in a poorer outcome. It is therefore pivotal that the healthcare provider not only understands and supports the child or adolescent but, at the same time, engages parents/caregivers to provide messages consistent with the assessment findings and management strategy of choice. From a healthcare provider’s perspective, it is important that when communicating clinical findings (their meaning and potential consequences), they are consistent with contemporary evidence. Importantly, patient education does not mean to educate the patient to the same professional level as the healthcare provider. Rather, it is to provide the child and caregivers with simple and individually tailored explanations that are aligned with, and supported by, contemporary science. Such an approach facilitates the transition from expert-driven management towards patient-centered care and supports patients and caregivers in making sense of the pain and provides them with tools to aid them in the self-management of recurrent, benign spinal pain.

## Figures and Tables

**Table 1:** An overview of important building blocks for designing a strategy that supports self-management in children and adolescents with spinal pain.

Steps	Suggestion for Implementation
Explain how pain works tailored to the individual and in alignment with contemporary evidence	Based on the assessment findings, explain how the individual’s perceived symptoms can be a result of a combination of biological processes (e.g., muscle fatigue) and the thoughts and beliefs we attach to it
2. Constructively address unhelpful health beliefs (the child’s and parents’)	Provide information on how our body becomes stronger from external load (e.g., a schoolbag) where the demands sometimes exceed what we are capable of. When this happens, we may experience discomfort that is not related to an injury
3. Promote reassurance regarding structural integrity of the spine	Our body is strong and is capable of amazing things. Even if we sustain our injury, it has the ability to heal and become as strong/stronger than before
4. Design and discuss an active management plan that builds on points 1 to 3	The plan needs to actively engage the individual and to some degree include the functions/activities in daily life that are challenging. Initially, this can be done under supervision, but focus should be on strategies that support independence and the ability to self-manage and adapt the recommended strategies to everyday functions

**Table 2:** An example of how the *Common Sense Model* can be used as a part of the assessment and management, if indicated by assessment findings.

Pain/Discomfort from Carrying the School Bag	Example of Lived Experience	Helpful Messaging	Unhelpful Messaging
What is this pain?	“My pain came when I walked to school with a heavy back”	“Your back/neck/shoulders become tired from carrying the schoolbag. When this happens, they become sore. However, this is not dangerous and over time you will adapt by making your body more resilient to carrying your bag”	“The schoolbag is too heavy for you. Because your body can’t handle such weight, you get pain”
What caused my pain?	“I don’t know what caused my pain. Maybe I walked too fast or perhaps my back pack was way too heavy?”	“Until you have built the resilience needed to carry your bag, your body needs to work extra hard for carrying the bag. This becomes better with training”	“Muscles and joints can’t sustain the load from the schoolbag and therefore become sore”
What are the consequences of my pain?	“It hurts and I think about it all the time. I don’t know what I can do to relieve it and am afraid I can make it worse if I walk too much or if I carry/lift heavy loads.”	“When you run for a long time, your legs get tired. The same happens to your back when you carry the schoolbag for a long time. The soreness does not mean that you risk damaging anything. If anything, you are making it stronger”	“Carrying the schoolbag despite being sore can result in the pain becoming chronic”
How well can I control my pain?	“There are a lot of things I used to be able to do without pain, but now it hurts when I walk with my backpack. I guess the only thing I can do is to stop walking and/or carrying my bag to school?”	“You can put the schoolbag down or change how you carry it when you get tired. You don’t have to carry it all day. This way, the soreness you may feel, will quickly go away when you get to school/home and can put the bag down.”	“The schoolbag is too heavy for you, but you have no choice but to carry it as you need all the books for school. There is no way around it”
How long will the pain last?	“I’m only 15 so I hope it doesn’t affect my plans for the future. I just don’t think the pain will go away if I have to continue doing what hurts me.”	“Carrying your schoolbag will eventually make the back stronger so you will get to a point where the weight won’t bother you anymore”	“You need to put up with this soreness until you no longer need to carry your schoolbag”
